# One-year surveillance for hypervirulent *Klebsiella pneumoniae* detected carbapenem-resistant superbugs

**DOI:** 10.1128/spectrum.03292-23

**Published:** 2024-01-30

**Authors:** C. Merla, A. Kuka, I. Mileto, G. Petazzoni, S. Gaiarsa, D. De Vitis, M. Ardizzone, M. Corbella, F. Baldanti, P. Cambieri

**Affiliations:** 1 Struttura Complessa di Microbiologia e Virologia, Fondazione IRCCS Policlinico San Matteo, Pavia, Italy; 2 Scuola di Specializzazione in Microbiologia e Virologia, Università degli Studi di Pavia, Pavia, Italy; 3 Dipartimento di Scienze Clinico-Chirurgiche, Diagnostiche e Pediatriche, Università degli Studi di Pavia, Pavia, Italy; University Paris-Saclay, AP-HP Hôpital Antoine Béclère, Service de Microbiologie, Institute for Integrative Biology of the Cell (I2BC), CEA, CNRS, France

**Keywords:** hypervirulent *Klebsiella pneumoniae*, NDM, multidrug resistance, KPC, ST23, ST86, ST147, ST6310

## Abstract

Hypervirulent *Klebsiella pneumoniae* (hvKp) can cause infections in clinically healthy people, such as young and immunocompetent patients. Genes involved in the capsule synthesis or those encoding the siderophores have been adopted as predictors of hvKp. Certain sequence types, such as ST23 and ST86, have been associated with hvKp strains, too. The aim of this study was to investigate the presence of hvKp among 354 *K. pneumoniae* strains isolated from clinical samples of patients admitted to an Italian 900-bed hospital between 21 May 2021 and April 2022. All the isolates were screened by PCR for the amplification of virulence loci. Whole genome sequencing was performed in strains tested positive for at least one target gene. Thirteen out of 354 (3.7%) were hvKp. Five were wild type and belonged to the hypervirulent clones ST23, ST86, ST5, and ST375 and to the new clone ST6310. Six strains carried the *blaKPC* gene: three belonged to ST101, two to ST512, and one to ST395. Two isolates were ST147 and carried the *blaNDM* gene. Although hvKp isolation is not frequent, their presence should be systematically investigated to avoid the spreading of both virulent strains and strains with combined increase in virulence and resistance to antibiotics. PCR-based protocols are essential for surveillance of these strains, which do not always show a recognizable phenotype. Moreover, hvKp strains were isolated also from patients without history of recent foreign travels, indicating an increased spreading of these strains as well as an underestimated of their circulation so far.

**IMPORTANCE:**

*Klebsiella pneumoniae* is a healthcare-associated pathogen frequently resistant to antibiotics. Hypervirulent strains of pneumoniae (hvKp) can spread from the primary site of infection to multiple sites causing life-threatening infections also in young otherwise healthy individuals. This study described the isolation of 13 isolates of *K. pneumoniae* with increased virulence in a large tertiary hospital over a 1-year period. Among them, eight strains were multidrug resistant and hypervirulent. Although these hypervirulent strains are still rare in Italy, their presence is particularly concerning since they can cause difficult-to-treat life-threatening infections. Moreover, not all the hypervirulent isolates were positive by the string test, so hvKp isolates were not always phenotypically detectable. Molecular biology techniques such as PCR amplification and next generation sequencing are therefore necessary for the detection of hvKp isolates, and surveillance programs exploiting molecular techniques are highly desirable.

## INTRODUCTION

*Klebsiella pneumoniae* has been recognized by the World Health Organization as a critical priority healthcare-associated pathogen due to antibiotic resistance. Strains of *K. pneumoniae* with increased virulence have spread from Asia, where the first strain with this characteristic was first isolated in the 1980s (1) to Europe and the United States (2–5).

Hypervirulent strains of *K. pneumoniae* (hvKp) can cause infections in young otherwise healthy individuals, differently from nosocomial multidrug resistant (MDR) *K. pneumoniae*. hvKp can disseminate from the primary site of infection to multiple sites causing life-threatening infections, such as hepatic abscesses, pneumonia, necrotizing fasciitis, endophthalmitis, meningitis, and sepsis (4).

Currently, the diagnosis of hvKp infections is based on clinical criteria, which includes unusual metastatic spread of *K. pneumoniae* infections, clusters of *K. pneumoniae* infections with increased severity and mortality. On the other hand, no single microbiological characteristic can define hvKp (6). Hypermucoviscosity is one of the main characteristics of hvKp strains, as most of these strains overexpress genes for capsular polysaccharides such as *rmpA* and *magA*, resulting in hypermucoid phenotype and higher resistance to phagocytosis and intercellular killing by neutrophils. Hypermucoviscosity is detected by string test, in which the colony is stretched with a loop. The formation of a viscous string longer than 5 mm is considered a positive string test (7). However, recent studies (8, 9) have shown that hypermucosity is not an exclusive feature of hvKp, while conversely, not all hvKp isolates exhibit a hypermucoviscous phenotype. Most of hvKp isolates belong to sequence types ST23, ST25, ST65, ST86, and ST375 (6).

The presence of iron acquisition systems (e.g., aerobactin, salmochelin, yersiniabactin) involved in bacterial growth and survival is indicative of hvKp strains (4, 6). Detection of genes such as *iutA* and *iroN*, which regulate iron acquisition systems, by PCR has been used in several studies (5, 10).

Recently, the *peg-344* gene has been included as a reliable, sensitive, and specific marker for the detection of hvKp such as aerobactin, salmochelin, *rmpA*, and *rmpA2* (8, 11). Indeed, PEG-344, whose function is still unclear, was proved to be required for maximal virulence in a pneumonia model (12).

Most hvKp strains remain susceptible to several commonly used antibiotics. However, carbapenem-resistant *K. pneumoniae* strains belonging to ST11 became hypervirulent by acquiring a pLVPK-like virulence plasmid encoding aerobactin, salmochelin, and RmpA (13). On the other hand, some hypervirulent strains of ST25 and ST65 exhibit carbapenem resistance (14, 15).

The presence of both increased virulence and resistance in carbapenem-resistant hvKp limits the number of effective antimicrobial agents and therefore poses a serious challenge to treatment, infection control, and public health.

The aim of this study is to investigate the presence of kvKp isolates among *K. pneumoniae* strains isolated over a period of 1 year from patients at Fondazione IRCCS Policlinico San Matteo in Pavia (Italy).

## MATERIALS AND METHODS

### Samples

Fondazione IRCCS Policlinico San Matteo is a 900-bed hospital, located in Lombardy region of Italy, where *Klebsiella pneumoniae* carbapenemase (KPC)- producing *K. pneumoniae* has been endemic since 2013 (16). In the study period, 1,822 *K. pneumoniae* isolates were collected during the routines of the bacteriology laboratory from a total of 627 patients. In this study, we searched for hvKp strains in 354 non-repetitive clinical *K. pneumoniae* isolates (one per patient) collected from patients at Fondazione IRCCS Policlinico San Matteo between May 2021 and April 2022. The selected 354 strains included strains with characteristics associated with virulence and strains from invasive infections such as bloodstream infections. In addition, we wanted to evaluate the presence of strains with both virulence and resistance characteristics that had never been reported in our hospital. Therefore, extended spectrum beta-lactamase (ESBL)-producing and carbapenem-resistant strains, hereafter referred to as MDR, were also considered. In detail, the 354 strains included:

- 21 *K. pneumoniae* strains positive by string test and isolated from any clinical specimen;
- 225 *K. pneumoniae* MDR strains negative by string test and isolated from any clinical specimen except for blood cultures; and
- 108 *K. pneumoniae* wild-type isolates obtained from blood cultures.

The type of specimens and the type of patient from which the 354 *K*. *pneumoniae* isolates have been isolated, as well as the resistance profile of the isolates, are shown in Table 1.

**TABLE 1:** Type of specimens and the type of patient from which the 354 *K*. *pneumoniae* isolates have been isolated^*a*^

Criterion of selection	Number of samples for the criterion	Inpatients	Outpatients	Type of specimen	Number of samples	Wild type	ESBL	KPC	NDM	VIM	Other
String test positive	21	13	8	Urine	13	7	5	1	NA^*b*^	NA	NA
				Blood culture	3	3	NA	NA	NA	NA	NA
				Respiratory samples	2	2	NA	NA	NA	NA	NA
				Liver biopsy	1	1	NA	NA	NA	NA	NA
				Rectal swab	1	NA	NA	1	NA	NA	NA
				Urethral swab	1	NA	NA	1	NA	NA	NA
String test negative and MDR	225	221	4	Rectal swab	160	NA	8	144	4	1	3 (2 KPC + VIM; 1 KPC + NDM)
				Respiratory samples	30	NA	6	24	NA	NA	NA
				Urine	25	NA	2	23	NA	NA	NA
				Wound/biopsy/dreinage samples	6	NA	NA	5	1	NA	NA
				Other	4	NA	NA	3	1	NA	NA
Blood cultures	108	80	28	Blood culture	108	57	19	30	1	NA	1 KPC + VIM

^*a*^ The resistance profile of the isolates is also shown.

^*b*^ NA was used to indicate that zero strains have that characteristic.

MALDI-TOF (Bruker Daltonics GmbH, Bremen, Germany) equipped with Bruker Biotyper 3.1 database was used for species identification. The antibiotic resistance profile was determined by the BD-Phoenix instrument (Becton Dickinson, USA), using the NMIC-505 panel.

### PCR amplification, sequencing, and genome analysis

Genomic DNA was extracted with a Blood and Tissue kit (QIAGEN, Düsseldorf, Germany) following the manufacturer’s instructions. PCR reactions were performed to investigate the presence of *iutA*, *iroN* loci (10), *rmpA* (17), and *peg-344* (18).

The genomes of the isolates positive for at least one virulence gene were sequenced. Short reads were obtained by Illumina MiSeq with a 2 × 150 paired-end run, after Nextera XT library preparation (Illumina Inc., San Diego, USA). Genomes were assembled using Shovill 1.1. Kleborate (19) was used to assess the sequence type and to search for virulence and resistance genes. The presence of plasmids was evaluated with PlasmidFinder (20) and manually curated.

A coreSNP-based phylogeny was performed for isolates belonging to ST147, since an outbreak caused by *K. pneumoniae* ST147 started in Tuscany region of Italy in 2018 and continued until 2021 (21). The data set consisted of 181 strains of *K. pneumoniae* ST147 from the outbreak (21, 22) and the two isolates from this study. A genomic background was constructed by retrieving the 20 most similar high-quality genomes (*N* = 94) to those in the data set from the BV-BRC database (https://www.bv-brc.org/) according to k-mer content similarity (MASH). The P-DOR pipeline (https://github.com/SteMIDIfactory/P-DOR) was used to align all genomes in the data set to a reference (573.4026 in BV-BRC) and extract coreSNPs. Maximum likelihood phylogeny was inferred with RAxML (using 100 bootstrap resamples) on the resulting coreSNP alignment using the general time reversible model, as suggested by ModelTest-NG, with ascertainment bias correction (23).

## RESULTS

*K. pneumoniae* was isolated from 627 patients during the study period. A total of 354 *K*. *pneumoniae* isolates were considered for this study, including 21 isolates with positive string test, 225 MDR isolates with negative string test, and 108 wild-type isolates from blood cultures with negative string test.

Twenty out of 354 isolates (5.6%) had at least one target gene of *rmpA*, *iutA*, *iroN*, and *peg-344* gene amplified, and six isolates had all four genes amplified. The characteristics of the 20 patients from whom the 20 isolates were obtained are shown in Table 2. The median age of the 20 patients was 64.35 years (range: 37–81), and 11 were female.

**TABLE 2:** Characteristics of the 20 patients from whom *Klebsiella pneumoniae* with increased virulence was isolated^*a*^

Strain number	Isolation date	Sample	Sex	Age	Hospital ward	Outcome	String test	Resistance mechanisms	PCR *rmpA*	PCR *iutA*	PCR *iroN*	PCR *peg-344*
5980	1May 2021	Rectal swab	F	74	General medicine	NA	−	KPC	−	+	−	+
5985	1 May 2021	Bronchial aspirate	F	63	Intensive care unit	Recovered	−	KPC	−	+	−	+
5999	6 May 2021	Bronchoalveolar lavage	M	74	Intensive care unit	Deceased	−	KPC	−	+	−	+
6002	8 May 2021	Rectal swab	M	52	Intensive care unit	NA	−	KPC	−	+	−	+
6007	12 May 2021	Rectal swab	M	56	Hematology	NA	−	KPC	−	+	+	−
6008	13 May 2021	Rectal swab	M	59	General medicine	NA	−	KPC	−	+	−	−
6082	8 June 2021	Liver biopsy	F	65	Intensive care unit	Deceased	+	Wild type	+	+	+	+
6114	22 June 2021	Urine	M	81	Infectious diseases (outpatients)	NA	+	KPC	−	+	−	−
hvKp_2	3 Augurst 2021	Bronchoalveolar lavage	M	44	Intensive care unit	Recovered	+	Wild type	+	+	+	+
hvKp_4	29 August 2021	Blood culture	F	71	Obstetrics and gynecology	Recovered	+	Wild type	+	+	+	+
6311	25 September 2021	Rectal swab	M	77	Intensive care unit	NA	−	KPC	−	+	+	−
6337	27 September 2021	Dreinage	F	49	Obstetrics and gynecology	Recovered	−	NDM	−	+	+	+
6452	21 October 2021	Blood culture	M	53	Intensive care unit	Recovered	+	Wild type	+	+	+	+
6399	27 October 2021	Rectal swab	F	75	Pneumology	NA	+	KPC	−	+	+	−
6403	27 October 2021	Rectal swab	F	76	Emergency room	NA	−	KPC	−	+	+	−
6445	11 November 2021	Rectal swab	M	37	Otolaryngology	NA	−	NDM	+	+	+	+
6465	12 November 2021	Urethral swab	F	76	Pneumology	Recovered	+	KPC	−	+	−	−
30715	24 November 2021	Blood culture	M	71	Intensive care unit	Deceased	+	Wild type	−	+	−	+
hvKp_19	24 November 2021	Urine	F	80	Outpatient sample collection center	NA	+	Wild type	+	+	+	+
6843	14 April 2022	Rectal swab	F	54	Pneumology	NA	−	KPC	−	+	+	−

^*a*^ Outcome for the patients colonized by *K. pneumoniae* with increased virulence is indicated with NA (Not Applicable).

Nine strains (2.54%) were isolated from rectal swabs and were resistant to carbapenems, with eight strains bearing *blaKPC* and one bearing *blaNDM* encoding for the New Delhi metallo-beta-lactamase (NDM). Only one isolate from rectal swabs was string test positive. The remaining 11 strains were isolated mainly from blood cultures (*n* = 3; 0.8%), bronchoalveolar lavage (BAL) (*n* = 2; 0.56%), and urine (*n* = 2; 0.56%). Four of 11 strains had KPC carbapenemase and one strain had NDM. The characteristics of all the 20 genomes are shown in Table 3.

**TABLE 3:** Characteristics of the genomes’ virulence score are attributed by Kleborate as follows: 0 = no yersinabactin, colibactin, or aerobactin; 1 = yersiniabactin only; 2 = yersiniabactin and colibactin (or colibactin only); 3 = aerobactin without yersiniabactin or colibactin; 4 = aerobactin with yersiniabactin (no colibactin); 5 = yersiniabactin, colibactin, and aerobactin^*a*^

Strain number	Sample	contig_count	N50	largest_contig	total_size	ST	virulence_score	resistance_score
5980	Rectal swab	163	203938	449385	5704928	ST512	0	2
5985	Bronchial aspirate	161	161807	544125	5712389	ST512	0	2
5999	Bronchoalveolar lavage	148	203931	449816	5708277	ST512	0	2
6002	Rectal swab	184	109995	334526	5847712	ST395	3	2
6007	Rectal swab	275	149647	331468	5702544	ST512	0	2
6008	Rectal swab	118	157687	405925	5560881	ST512	1	3
6082	Liver biopsy	100	195392	498479	5445960	ST375	3	0
6114	Urine	140	158510	449810	5601209	ST512	0	2
hvKp_2	Bronchoalveolar lavage	115	116031	303700	5590050	ST6310	3	0
hvKp_4	Blood culture	85	316230	436906	5473727	ST86	4	0
6311	Rectal swab	131	202114	426621	5757831	ST101	4	3
6337	Dreinage	179	219779	823629	5748271	ST147	3	2
6452	Blood culture	116	246519	788521	5655516	ST23	5	0
6399	Rectal swab	202	136210	489606	5939332	ST512	3	2
6403	Rectal swab	164	205081	380663	5818972	ST101	4	3
6445	Rectal swab	228	173045	386555	5930012	ST147	4	2
6465	Urethral swab	155	201170	523991	5941613	ST512	3	2
30715	Blood culture	109	244625	449964	5425355	ST727	1	0
hvKp_19	Urine	61	323151	680484	5437774	ST5	4	0
6843	Rectal swab	172	205082	520390	5820568	ST101	4	3

^*a*^ Resistance score is attributed by Kleborate as follows: 1 = ESBL; 2 = carbapenemase; 3 = carbapenemase plus colistin resistance; 0 no resistance mechanism to beta-lactams.

Kleborate analyses showed that 13 (3.64%) strains had virulence score above 3 and seven of them were string test positive (Table 4). One strain isolated from a blood culture belonged to ST23 and had the highest virulence score. This strain is the only one in this study displaying aerobactin, salmochelin, yersiniabactin, and colibactin.

**TABLE 4:** Characteristics of the 20 strains that were sequenced^*a*^

Strain	String test	Resistance mechanisms	ST	Virulence score	Yersiniabactin	Colibactin	Aerobactin	Salmochelin	RmpADC	rmpA2
5980	−	KPC	ST512	0	−	−	−	−	−	−
5985	−	KPC	ST512	0	−	−	−	−	−	−
5999	−	KPC	ST512	0	−	−	−	−	−	−
6002	−	KPC	ST395	3	−	−	iuc 1	−	−	−
6007	−	KPC	ST512	0	−	−	−	−	−	−
6008	−	KPC	ST512	1	ybt 9; ICEKp3	−	−	−	−	−
6082	+	Wild Type	ST375	3	−	−	iuc 1	iro 1	rmp 1; KpVP-1	rmpA2_3-47%
6114	+	KPC	ST512	0	−	−	−	−	−	−
hvKp_2	+	Wild Type	ST6310	3	−	−	iuc 1	iro 1	rmp 1; KpVP-1 (truncated)	−
hvKp_4	+	Wild Type	ST86	4	ybt 9; ICEKp3	−	iuc 1	iro 1	rmp 1; KpVP-1	rmpA2_9
6311	−	KPC	ST101	4	ybt 9; ICEKp3	−	iuc 1	−	−	−
6337	−	NDM	ST147	3	−	−	iuc 1	−	rmp 1; KpVP-1	rmpA2_6*−47%
6452	+	Wild Type	ST23	5	ybt 1; ICEKp10	clb 2	iuc 1	iro 1	rmp 1; KpVP-1	rmpA2_5-54%
6399	+	KPC	ST512	3	−	−	iuc 1	−	−	−
6403	−	KPC	ST101	4	ybt 9; ICEKp3	−	iuc 1	−	−	−
6445	−	NDM	ST147	4	ybt 9; ICEKp3	−	iuc 1	−	rmp 1; KpVP-1	rmpA2_6*−47%
6465	+	KPC	ST512	3	−	−	iuc 1	−	−	−
30715	+	Wild Type	ST727	1	ybt 4; plasmid (incomplete)	−	−	iro 1	rmp unknown	−
hvKp_19	+	Wild Type	ST5	4	ybt 2; ICEKp1	−	iuc 3 (truncated)	iro 3 (truncated)	rmp 3; ICEKp1 (truncated)	−
6843	−	KPC	ST101	4	ybt 9; ICEKp3	−	iuc 1	−	−	−

^*a*^ Resistance mechanism, sequence type, and the presence of virulence loci are displayed. A negative string test is indicated with “−”, while a positive one is indicated with “+”.

Eight strains meet the definition of convergent antimicrobial resistant and virulent strains as proposed by Lam et al. (19), having virulence score ≥3 and resistance score ≥1 (Table 3). Three strains belonging to ST101 isolated from rectal swabs and one strain ST147 isolated from a rectal swab had a virulence score of 4 and had aerobactin, salmochelin, and yersiniabactin. The three strains ST101 were KPC producing and the strain ST147 was NDM producing. The other four convergent antimicrobial resistant and virulent strains had a virulence score of 3 and belonged to ST147, ST395, and ST512.

**Fig 1:**
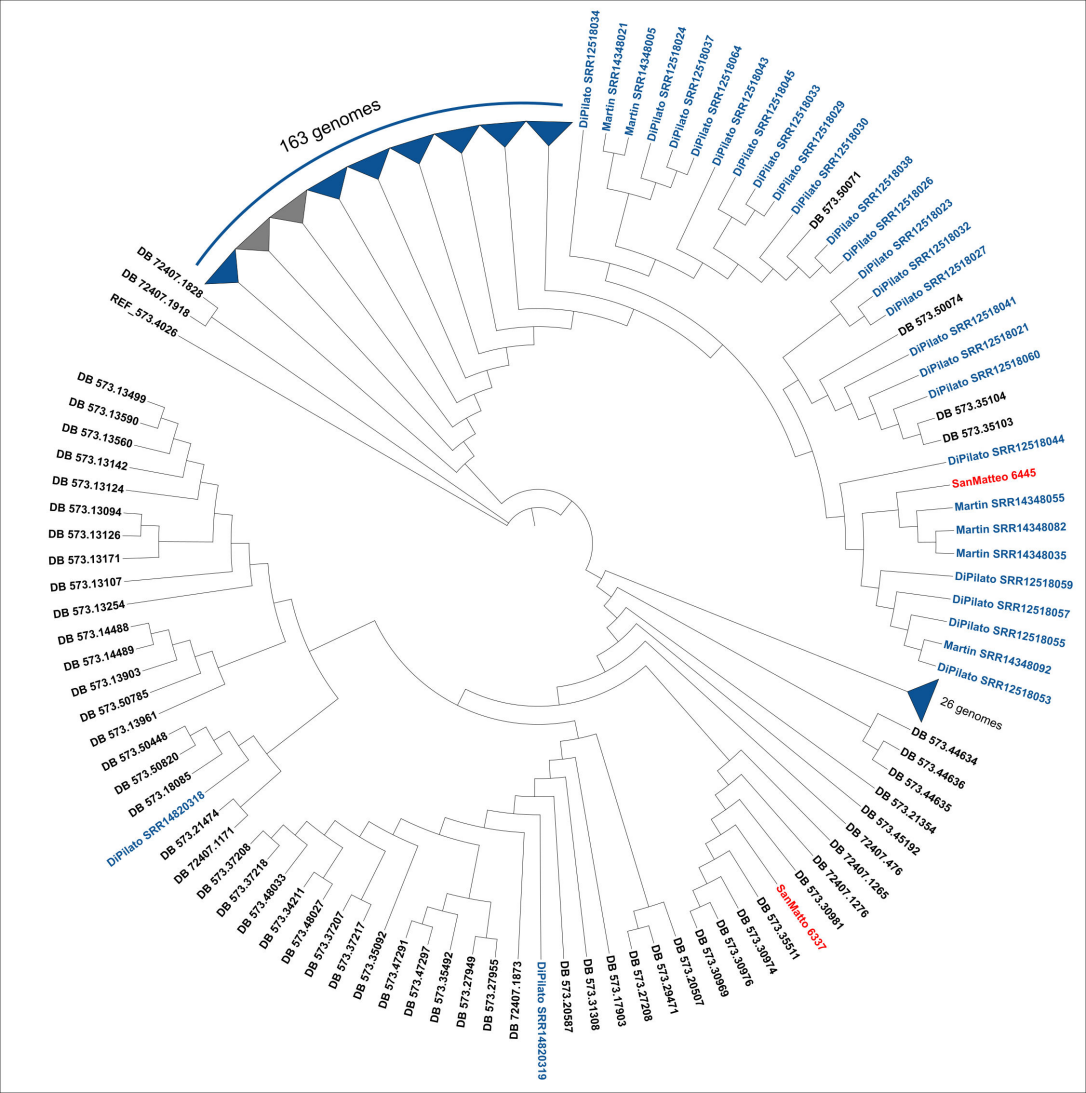
Maximum likelihood phylogeny of *Klebsiella pneumoniae* ST147 based on SNP cores obtained using the P-DOR pipeline (https://github.com/SteMIDIfactory/P-DOR). The two strains of this study are indicated in red, while 181 genomes ST147 from the outbreak that occurred in Tuscany reported by Martin et al. (21) and Di Pilato et al. (22) are in blue.

A phylogeny that included the two strains ST147 isolated in this study and the 181 ST147 strains from the outbreak that occurred in Tuscany (21, 22) is shown in Fig. 1. One of the two strains of this study (SanMatteo_6445) clustered in a monophyletic clade with three other strains from the study by Martin et al. (21) and the common branch is highly supported (100/100 bootstraps). Evaluation of clonal relatedness by core-genome SNPs reveals that clones were overall highly related to the cluster (SNP range: 19–20). As for other strains in the same cluster, the FIB(pQil)-type plasmid (pQil-NDM-147Tu; 56,064 bp; GenBank accession number CP071030) was identified. This plasmid carried the *bla_NDM-1_* gene, along with other resistant determinants such as *aacA4*-cr, *bla_OXA-1_*, and *bla_CTX-M-15_*. In addition, the HIB-FIB(Mar) plasmid (pVIR-147Tu; 341,914 pb; GenBank accession number CP071028) was found. This one carried several resistant determinants such as *armA*, *sul1* and *sul2*, *bla_CTX-M-15_*, and virulence ones such as *rmpADC*, *iucABCD-iutA*, and *peg-344*. On the contrary, the other strain (SanMatteo_6337) clustered with high branch support (94/100 bootstraps) with four strains from the BV-BRC DB isolated in Russia between 2018 and 2019 (SNP range: 41–104). The SanMatteo_6337 strain did not carry the FIB(pQil)-type plasmid and carried the HIB-FIB(Mar)-type plasmid, instead.

## DISCUSSION

This study reports the isolation of 13 isolates of hypervirulent *K. pneumoniae,* based on sequence type and the presence of virulence genes such as aerobactin and salmochelin. Six out of 13 strains were isolated from rectal swab (colonization), while seven strains caused infections.

The 13 strains had different characteristics. Two strains belonged to ST23 and hvKp ST86, respectively, and caused bloodstream infections in two patients who had not recently traveled abroad. hvKp ST23 was reported for the first time in Italy in 2014 (24), and in our hospital, it was isolated from urine and BAL samples between 2017 and 2018 (25). However, this sequence type is very uncommon in Italy and only five genomes of *K. pneumoniae* ST23 were found in the BV-BRC database as isolated in Italy, including the 6453 isolate of this study and the isolate from a BAL sample of the same patient (last accessed on 30 March 2023). ST23 strain isolated in this study was wild type but hvKp ST23 bearing carbapenemases were described in several European countries also from blood and respiratory samples (3, 24).

Equally rare is the ST86, which presence is documented in Italy so far only by the presence in BV-BRC databases of three genomes of *K. pneumoniae* ST86, including strain hvKp_4 of this study (reported in the BV_BRC database as 10028588). In Europe, ST86 hvKp strains have already been reported in Spain where three isolates out of 878 were found to be hypervirulent ST86 in a 7-year study (2) and in France where three out of 59 *K*. *pneumoniae* isolates were hypervirulent ST86 in a 5-year study (3). In France, carbapenem-resistant hvKp bearing OXA-48 have been isolated as well (26).

Other sequence types frequently associated with hypervirulence, such as ST5 and ST375, were found in this study. ST5 was previously isolated in Italy in a multicentric study (27). This strain, as well as hvKp_19 of this study, bears virulence factors such as ICEKp1, aerobactin, salmochelin, and rmpADC hypermucoidy locus. Only four genomes belonging to ST375 were found in the BV-BRC database as isolated in Europe, none in Italy. Hypervirulent ST375 was previously isolated in Japan (28) from liver abscesses of two patients. The ST375 isolate of this study was isolated from a liver biopsy, too, and as the isolates from Japan, it carried aerobactin and *rmpA* genes.

The new sequence type ST6310, a single locus variant of ST25 for *mdh* gene, was described for the first time in this study. Strain hvKp_2, which belonged to ST6310, had aerobactin, salmochelin, and *rmpA* loci and it was isolated from a patient who had lived in China.

hvKp isolates belonging to well-known MDR high-risk clones such as ST101, ST147, ST395, and ST512 were also found in this study. hvKp are usually susceptible to most antibiotics, but the ability of *Klebsiella* to acquire genetic elements leads to the emergence of MDR-hvKp clones (4, 13). Most MDR strains of this study did not have multiple virulence genes, except for three strains belonging to ST101 and one from ST147 that bore a wider range of virulence genes. The latter ST147 strain resulted closely related to the strains that caused a large hospital outbreak in the Tuscany region of Italy. Indeed, this isolate along with the strains described in the study of Di Pilato et al. are part of subclade of ST147, called ST147-vir, characterized by the presence of the *ybt*-encoding element ICEKpn3 and the pQil-NDM-147Tu-like and the pVIR-147Tu-like plasmids (22). The sequence type 147 is a high-risk clone associated with hospital-acquired infections worldwide and mediating the global spread of NDM-like metallo-beta-lactamase genes (29). Therefore, it is crucial to track the dissemination of these potential successful clones in hospital settings and understand their evolution to avoid the spread of resistant and virulent determinants, especially those determinants carried on plasmids, such as *bla_NDM-1_*.

In conclusion, this study described the isolation of 13 isolates of *K. pneumoniae* with increased virulence in a large tertiary hospital over a 1-year period. Although the isolation of these strains is not frequent, their presence is particularly concerning, as some of these isolates are not only hypervirulent but also MDR, effectively limiting the therapeutic options in case of infections.

Not all the hypervirulent isolates were positive by the string test, so hvKp are not always phenotypically detectable. Molecular biology techniques such as PCR amplification and next generation sequencing are therefore necessary for the detection of hvKp isolates. Surveillance programs exploiting PCR for the detection of hvKp isolates are desirable also because virulence genes are often carried on mobile genetic elements, which can be transferred within and between different bacterial species. In addition, the presence of hvKp should be systematically investigated in both wild-type and MDR strains, to avoid the spreading of strains with both increased virulence and resistance to antibiotics. Finally, hvKp strains were also isolated from patients with no history of recent travels abroad, suggesting an increased prevalence or an undetected circulation of hvKp isolates.

## Data Availability

Genome assembly data are available at NCBI under BioProject ID PRJNA814467.

## AFTER EPUB

*[This article was published on 30 January 2024 with inaccurate information in the legend of Fig. 1. The legend was updated in the current version, posted on 22 February 2024.]*
